# Optimization of Alumina Ceramics Corrosion Resistance in Nitric Acid

**DOI:** 10.3390/ma15072579

**Published:** 2022-03-31

**Authors:** Ivana Ropuš, Lidija Ćurković, Hrvoje Cajner, Sanda Rončević

**Affiliations:** 1 Energoatest Zaštita d.o.o., Potočnjakova 4, HR-10000 Zagreb, Croatia; ivanaropus@gmail.com; 2 Department of Materials, Faculty of Mechanical Engineering and Naval Architecture, University of Zagreb, HR-10000 Zagreb, Croatia; 3 Department of Industrial Engineering, Faculty of Mechanical Engineering and Naval Architecture, University of Zagreb, HR-10000 Zagreb, Croatia; 4 Department of Chemistry, Faculty of Science, University of Zagreb, Horvatovac 102a, HR-10000 Zagreb, Croatia; sanda.roncevic@chem.pmf.hr

**Keywords:** alumina ceramic, corrosion, response surface methodology

## Abstract

The development of ceramic materials resistance in various aggressive media combined with required mechanical properties is of considerable importance for enabling the wider application of ceramics. The corrosion resistance of ceramic materials depends on their purity and microstructure, the kind of aggressive media used and the ambient temperature. Therefore, the corrosion resistance of alumina ceramics in aqueous HNO_3_ solutions of concentrations of 0.50 mol dm^−3^, 1.25 mol dm^−3^ and 2.00 mol dm^−3^ and different exposure times—up to 10 days—have been studied. The influence of temperature (25, 40 and 55 °C) was also monitored. The evaluation of Al_2_O_3_ ceramics corrosion resistance was based on the concentration measurements of eluted Al^3+^, Ca^2+^, Fe^3+^, Mg^2+^, Na^+^ and Si^4+^ ions obtained by inductively coupled plasma atomic emission spectrometry (ICP-AES), as well as density measurements of the investigated alumina ceramics. The response surface methodology (RSM) was used for the optimization of parameters within the experimental “sample-corrosive media” area. The exposure of alumina ceramics to aqueous HNO_3_ solutions was conducted according to the Box–Behnken design. After the regression functions were defined, conditions to achieve the maximum corrosion resistance of the sintered ceramics were determined by optimization within the experimental area.

## 1. Introduction

Alumina (Al_2_O_3_) is a ceramic material that possesses high values of hardness, strength and wear resistance, as well as chemical stability [1,2,3,4]. Therefore, it may be applied as an advanced material in electronics, metallurgy, catalysis, wear protection, refractories, as a composite, etc. [5,6,7]. Nevertheless, the issue of ceramic corrosion is considered and investigated in many fields, such as geochemical research, nuclear waste disposal, art history and archaeological research—including industrial applications [8,9]. Small amounts of impurities and additives have a considerate impact on the production and the final properties of alumina-based ceramics [10]. Grain boundaries of alumina ceramics are sensitive to chemical attacks, which can consequently cause changes in the alumina corrosion resistance. An increase of control over the grain boundary chemistry of polycrystalline alumina may lead to the production of polycrystalline alumina that has a structure comparable to a single crystal sapphire, which is considered to be a highly corrosion resistant material because of the absence of grain boundaries [10,11].

Ceramic corrosion is based on different corrosion mechanisms compared to metal corrosion. The corrosion of metals is mostly a consequence of electrochemical processes, while the corrosion of ceramics is a result of dissolution in different media [12,13]. With rapid development and, accordingly, the new application possibilities of advanced ceramics, the demand for more knowledge of their chemical resistance in aggressive acid environments has increased significantly.

Miyashita et al. [13] investigated the corrosion resistance of dense Y_2_O_3_, YOF, Y_5_O_4_F_7_ and Y_5_O_4_F_7_ + YF_3_ ceramics in 3.00 mol dm^−3^ HCl, HNO_3_ and HF solutions at room temperature. They pointed out that Y_2_O_3_, which is slightly alkaline, is less corrosion resistant to HCl and HNO_3_ than YOF, Y_5_O_4_F_7_ and Y_5_O_4_F_7_ + YF_3_, because of acid–base reactions. However, all of the samples showed a higher corrosion resistance to HF than to HCl and HNO_3_ solutions. These conclusions were made according to weight loss measurements. Furthermore, intergranular attacks and damage to the ceramic surfaces were observed by SEM microstructure analysis. Mikeska et al. [11] investigated the chemical stability of commercially available oxide ceramics (Al_2_O_3_, TiO_2_ and ZrO_2_) and non-oxide ceramics (Si_3_N_4_, AlN, BN, SiC, TiC, B_4_C and WC) in hydrofluoric acid (HF) at 90 °C in a time frame of up to two weeks by observing their weight change and microstructure. Among the observed ceramics, they found polycrystalline carbides to be the most corrosion resistant. Furthermore, they confirmed an increase of chemical stability of commercial alumina with an increase in their purity. Consequently, sapphire was shown as the most stable alumina in HF after two weeks of exposure, followed by 99.9% pure alumina. Alumina corrosion resistance to acidic and base solutions was reported in the literature [2,3,5]. Schacht et al. [2] also identified certain attacks of acidic aqueous solutions at high temperatures and pressures to the grain boundaries. In some cases [4,11,14,15], the chemical stability was measured by monitoring the weight loss and mechanical properties after exposure to the corrosive media. When the weight loss of ceramic material is below the detectability of the analytical balance, the corrosion can be determined by measuring the number of eluted ions in corrosive media [16]. Börensen et al. [17] observed in situ formations of nitrate, water and intermediate nitrite molecules in the reaction of NO_2_ and HNO_3_ with the alumina surface. They concluded that the nitrate formation on mineral aerosol from the NO_2_ reaction would be negligible. Bennet [18] exposed commercial ceramic materials, including alumina (85 wt% Al_2_O_3_) for 110 days in HCl, HNO_3_ and H_2_SO_4_. Acid concentrations ranged from 10 to 90 wt% and temperatures ranged from 50 °C to 90 °C. The leaching of Al and Fe ions was higher than for the rest of the monitored ions (Ca, Fe, K, Mg, Na, Si and Ti). The investigated alumina had a higher corrosion resistance to H_2_SO_4_ than to HCl and HNO_3_.

The examination of the parameters influencing ceramic corrosion can be accomplished by the “one-factor-at-the-time” approach (OFAT), which is a time-consuming approach. OFAT is also incapable of reaching a true optimum because it does not consider interactions among factors. On the contrary, response surface methodology (RSM) is a useful tool for examining the existence of these interactions between the factors of the process and, subsequently, optimizing it [19].

Considering the complexity of the impact of different factors on the corrosion process of ceramics, the need for the development of a model that could determine interactions between factors, predict the development of corrosion processes within experimental areas and define the conditions for minimal corrosion, is evident. This kind of model could also significantly lower the maintenance costs and extend the life expectancy of ceramic materials, i.e., alumina, within the given conditions.

In this study, Box–Behnken design was applied to study the impact of immersion time, temperature and concentration of nitric acid (HNO_3_) on the chemical stability of sintered alumina samples by monitoring their density and the amount of eluted ions (Al^3+^, Ca^2+^, Fe^3+^, Mg^2+^, Na^+^ and Si^4+^) from the samples during the static corrosion test.

## 2. Materials and Methods

### 2.1. Preparation of Al_2_O_3_ Ceramics

The chemical composition of the raw used alumina (produced by Alteo, Gardanne, France) is shown in Table 1.

Alumina granules, produced by a spray drying process, were isostatically cold shaped into cylindrical (green) compacts at Applied Ceramics Inc., Sisak, Croatia. Each green compact was engraved with a number, in order to follow the properties of each one during the experiment. Green compacts were sintered in a high-temperature furnace P310 (Nabertherm, Lilienthal, Germany) using the following regime: initial heating at a rate of 5 °C min^−1^ up to a temperature of 500 °C, holding at 500 °C for 30 min, further heating at a rate of 5 °C min^−1^ up to 1600 °C, holding at 1600 °C for 6 h and slow cooling in the furnace to room temperature.

### 2.2. Characterisation of Alumina Ceramics

The phase composition of Al_2_O_3_ granules was determined by powder X-ray diffraction, PXRD (Shimadzu XRD6000, Shimadzu Corporation, Kyoto, Japan) with CuKα radiation. The step size of 0.02 degrees between 10° and 80° 2θ and a counting time of 0.6 s were used, under an accelerating voltage of 40 kV and a current of 30 mA.

The morphology of the prepared sintered samples was determined according to standard ceramographic technique [20] by means of scanning electron microscope (SEM) (Tescan Vega TS5136LS, Prague, Czech Republic).

The bulk density of the sintered alumina samples was determined by the Archimedes method (Mettler Toledo GmbH, Greifensee, Switzerland, density kit MS-DNY-43) according to ASTM C373-88. 

The relative density of the sintered samples was calculated by the following equation:

(1) $$\rho_{relative}=\frac{\rho_{Archimeds}}{\rho_{theoretical}}\;\cdot 100\%$$

while the relative porosity is calculated as a difference between 100% density and relative density (%) [21].

The hardness of the sintered samples was measured by means of the hardness tester Wilson Wolpert Tukon 2100B (Instron, Grove City, PA, USA). Diagonals were measured by optical microscope Olympus BH (Olympus Imaging Corp., Tokyo, Japan) immediately after unloading. Vickers hardness was measured 10 times per sample.

Fracture toughness was determined after Vickers’s indentation. The ratio of the crack length and half of the indentation diagonal (*c/a*) indicates the crack type, which is used as an indirect indicator of the ceramic toughness [6,22,23,24]. Care was taken to make indentations only on those areas that had no visible pores. Furthermore, indentation points were randomly chosen over the polished surfaces with a sufficient distance between indentation spots in order not to impact the crack growth of the ceramics during testing. The crack dimensions were not allowed to exceed one-tenth of the thickness of the samples [25].

Although the crack growth of the sintered alumina may be influenced by, e.g., temperature field and thermal stress [26], these effects will not be explored in this research. The research provides measurements that were obtained at ambient temperature.

### 2.3. Corrosion Monitoring of Alumina in Aqueous HNO_3_ Solution

The sintered alumina samples were cleaned with alcohol and dried in a sterilizer at 150 ± 5 °C for 4 h. Polypropylene (PP) tubes were marked and filled with 10 cm^3^ of the adequate concentration of HNO_3_. The samples were then immersed into the acid solutions and the PP tubes were sealed. The concentrations of HNO_3_ used in this experiment were 0.50, 1.25 and 2.00 mol dm^−3^. A static corrosion test was carried out according to the Box–Behnken design at 25, 40 and 55 °C. The factors and design points are shown in Table 2 and Table 3.

Afterwards, the alumina samples were removed from the tubes, rinsed with distilled water and dried in an oven for 3 h at 150 °C. Finally, the bulk density of the alumina samples after the corrosion test was measured.

During the corrosion testing, the weight of the alumina samples remained unchanged (measured on an analytical balance with a precision of 10^−5^ g). The mechanisms responsible for the corrosion processes were observed by determining the concentration of ions (Al^3+^, Ca^2+^, Fe^3+^, Mg^2+^ and Na^+^ ions) eluted into the corrosive aqueous HNO_3_ solution. The concentration of eluted ions was determined by ICP—AES, Teledyne Leeman Labs (Hudson, NH, SAD). The Si^4+^ cations were under the quantification limit (LOQ (Si^4+^) < 0.45 µg/g) [27].

### 2.4. Design of Experiments of Monitoring Alumina Corrosion Resistance

The Box–Behnken design was applied to avoid experiments performed under extreme conditions (vertices of the cube), where unsatisfactory results might occur [19]. The number of experiments also decreased compared to the other designs of RSM [28], which is beneficial in terms of time and other resource limitations (materials and equipment).

According to previous studies [5,7,16,29,30], three factors (input variables) that impact the chemical stability of ceramics in acidic solutions were selected: temperature, concentration and immersion time in HNO_3_. Each factor was varied at three levels with five replicates, which were conducted at the center point (Table 2 and Table 3).

Design Expert^®®^ software (version 13) by Stat-Ease Inc. (Minneapolis, MN, USA) was used to model and analyze the causal relationship between the input and output variables and to perform the diagnostic analysis as well. Calculated regression models provided the quantification of the temperature, time and corrosive media concentration (aqueous HNO_3_ solutions) effects on alumina density and the number of eluted ions. It must be noted that reported models were applicable only in the range defined by the experimental area (Table 2). Subsequently, six response variables were measured: density of the investigated alumina ceramics and the amount of Al^3+^, Ca^2+^, Fe^3+^, Mg^2+^ and Na^+^ ions eluted from Al_2_O_3_.

## 3. Results and Discussion

### 3.1. Properties of Alumina

**Figure 1:**
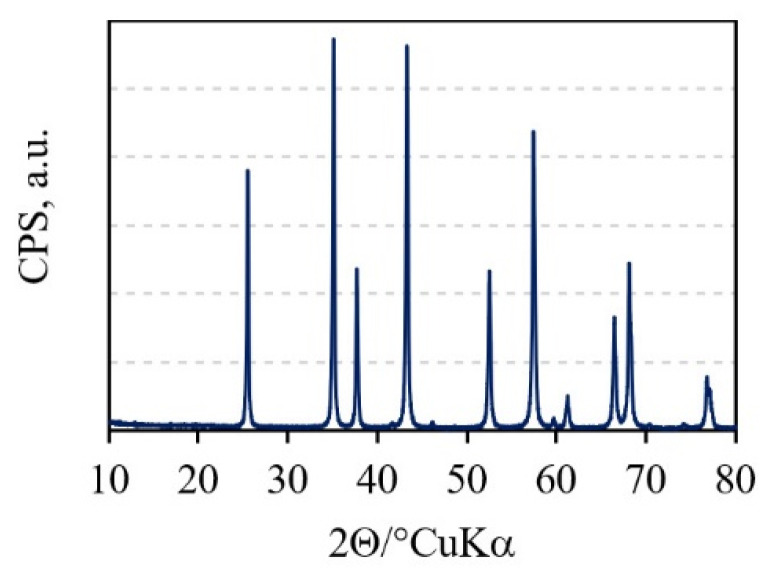
XRD pattern of the Al_2_O_3_ granules.

The X-ray diffractogram (Figure 1) of the alumina granules showed the presence of the characteristic peaks of the only phase that was α-Al_2_O_3_.

**Figure 2:**
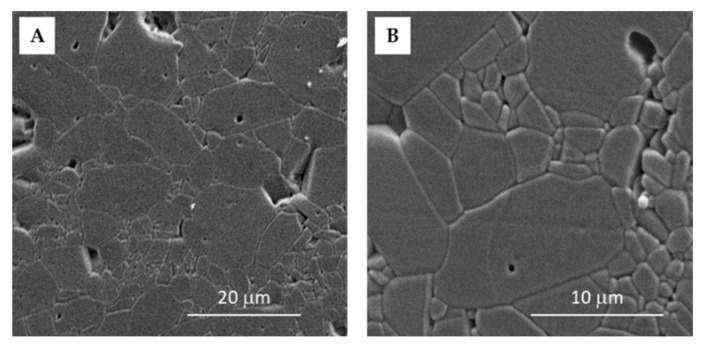
SEM images of the sintered Al_2_O_3_ ceramics with the magnification of (**A**) 2500× and (**B**) 6000×.

During sintering, grains and grain boundaries were formed. The competition between coarsening and densification during the 6 h of sintering led to the formation of nonuniform grains in size (cca 0.7–8 µm) and orientation. The average grain size was 7.6 µm (Figure 2), which was calculated through the line intercept method [31,32] and is in accordance with the literature data [7].

The measured bulk density was 3.864 ± 0.018 g cm^−3^, while the relative porosity was 3.1 ± 0.5%. The mechanical properties, such as hardness and fracture toughness, are given in Table 4. Vickers hardness was measured at a load of 9.807 N.

Cracks obtained during the hardness measurement indicated the Palmqvist crack system while the *c/a* ratio was less than 2.5 [33]. Fracture toughness *(K*_IC,_ MPa m^1/2^) was determined according to Casellas [23,24,34,35]:

(2) $$K_{\mathrm{Ic}}=0.024\cdot \frac{F}{c^{1.5}}\cdot {\left(\frac{E}{HV}\right)}^{0.5}$$

where *F* is applied load, N; *c* half-length of crack, m; *E* is Young’s modulus, GPa; and HV is Vickers hardness.

### 3.2. Modeling of the Amount of Eluted Ions and Alumina Density

As described, the corrosion test was conducted for sintered alumina to determine their corrosion resistance to three concentrations of HNO_3_ in a time frame of up to 10 days at different temperatures.

The results of the analysis of variance (ANOVA) of the obtained data regarding the amounts of eluted ions and Al_2_O_3_ sample density showed the statistical significance of each factor. The ANOVA table for the amount of Al^3+^ eluted ions is given in Table 5. 

**Figure 3:**
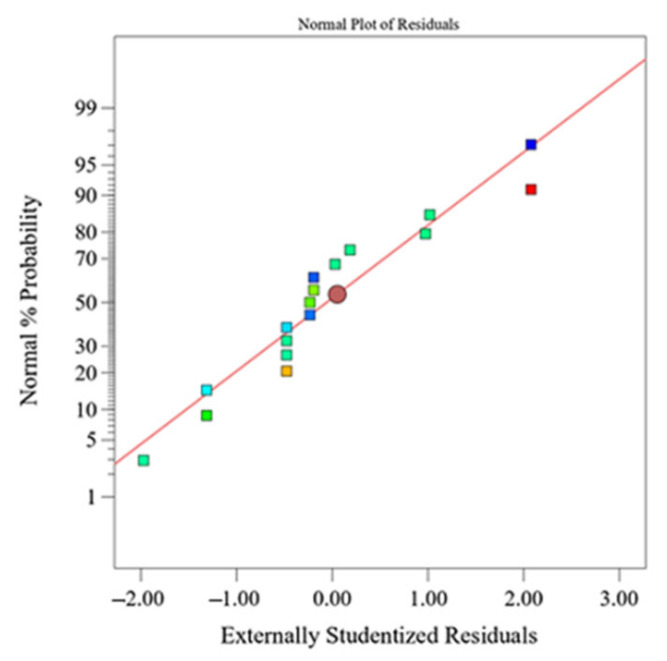
Normal plot of response residuals—amount of eluted Al^3+^ ions from Al_2_O_3_ ceramics after exposure to HNO_3_.

The regression models explained more than 98% of the total variation of the amount of all eluted ions and more than 83% of the density variation (according to the determination coefficient, *R*^2^). The normal probability plots for the eluted ions, as well as the one for alumina density, have a similar behavior compared to the normal plot of the amount of eluted Al^3+^ ions, which is shown in Figure 3. The normal plot of the residuals shows that there is no significant and undesirable trend, which indicates a normal distribution of residuals [36,37].

**Figure 4:**
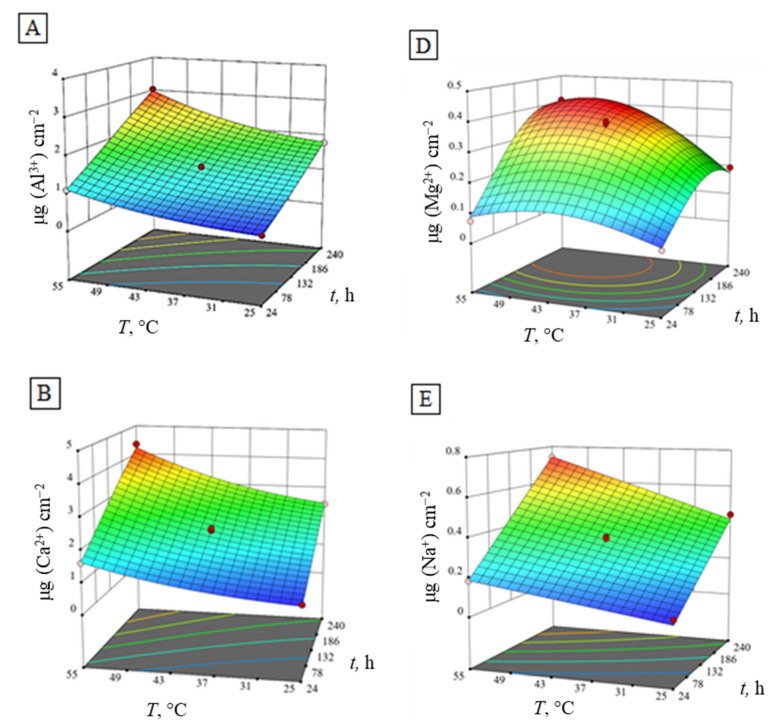

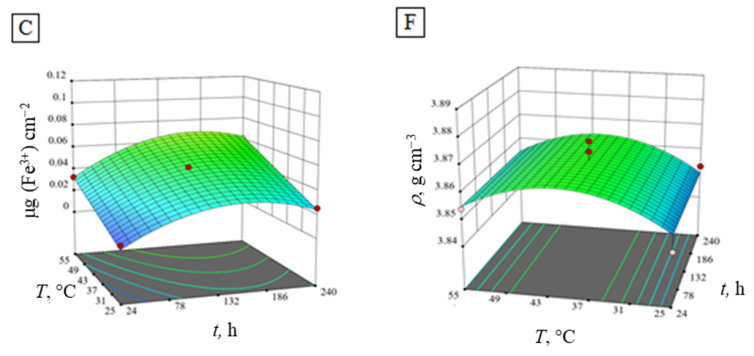
Response surface plots of the regression models of the amount of eluted ions (**A**) Al^3+^, (**B**) Ca^2+^, (**C**) Fe^3+^, (**D**) Mg^2+^, (**E**) Na^+^ and (**F**) density of Al_2_O_3_ at a constant concentration (1.25 mol dm^−3^) of HNO_3_.

High *R*^2^ values and a normal distribution of response residuals demonstrated the adequacy of the obtained models [38]. In Table 6, the experimental data used for the calculation of the regression equations are presented. Response surface plots, as graphic representations of the regression models, are shown in Figure 4.

### 3.3. Optimization and Verification of Alumina Ceramics Corrosion Resistance in Nitric Acid

The optimum values of the selected independent variables were obtained using numerical optimization and by analyzing the response surface plots (graphical optimization). Models generated by RSM were verified by conducting experiments at the numerically obtained optimized parameters. Experimentally obtained results and results predicted by the model were compared to evaluate the accuracy and suitability of the model. The applicability of the regression models, listed in Table 7, were tested and confirmed by conducting five verification points. All of the results fall within 95% of the confidence interval of the mean, which also proves that it satisfies the 95% of prediction interval, leading to the conclusion that the models are applicable and useful for predicting the values of the responses.

Figure 4A,B,E shows similar response surface plots of the regression models for the amount of eluted Al^3+^, Ca^2+^ and Na^+^ ions in HNO_3_ during the experiment at a constant concentration of HNO_3_ (1.25 mol dm^−3^). Contrary to that, the eluted Fe^3+^, Mg^2+^ ions and alumina density (Figure 4C,D,F) show more convex shaped response surface plots. With the increase of HNO_3_ temperature and time, the increase of all eluted ions is evident. However, time is not shown as a statistically significant factor for the regression model, even though in practice, the impact of time is notable [39]. Maximum values of eluted Al^3+^, Ca^2+^ and Na^+^ ions in HNO_3_ are reached at the highest temperature and longest immersion time in HNO_3_, while the maximum amount of eluted Fe^3+^ and Mg^2+^ ions, as well as alumina density peaks, are achieved earlier, at lower temperatures. Conclusively, the number of eluted ions from the alumina ceramics obtained from the corrosion experiments are in the following order:

Fe^3+^ < Mg^2+^ < Na^+^ < Al^3+^ < Ca^2+^

The corrosion resistance of alumina ceramics is influenced by the purity of the material due to the segregation of impurities to the grain boundaries during the sintering process. The presence of SiO_2_, at concentrations above cca 1000 ppm, is detrimental due to the formation of a silicate-rich glassy phase on the grain boundaries, which is easily attacked by mineral acids [2]. Alumina ceramics corrosion is in correlation with their microstructure and distribution of CaO, Fe_2_O_3_, MgO, Na_2_O and SiO_2_ in it. The distribution and solubility of CaO, Fe_2_O_3_, MgO, Na_2_O and SiO_2_ in alumina ceramics depends on the difference in the charge and ionic radius of Ca^2+^ (100 pm), Fe^3+^ (64.5 pm), Mg^2+^ (72 pm), Na^+^ (102 pm) and Si^4+^ (40 pm) compared to the Al^3+^ (53.5 pm) cation [40]. When cations are not soluble in the crystal lattice of alumina ceramics, they segregate to the grain boundaries [2,16]. Therefore, impurities in alumina ceramics, such as CaO, Fe_2_O_3_, Na_2_O and SiO_2_ and sintering aid MgO, have a low solubility in Al_2_O_3_ and move to the grain boundaries during the sintering process, where they segregate.

**Figure 5:**
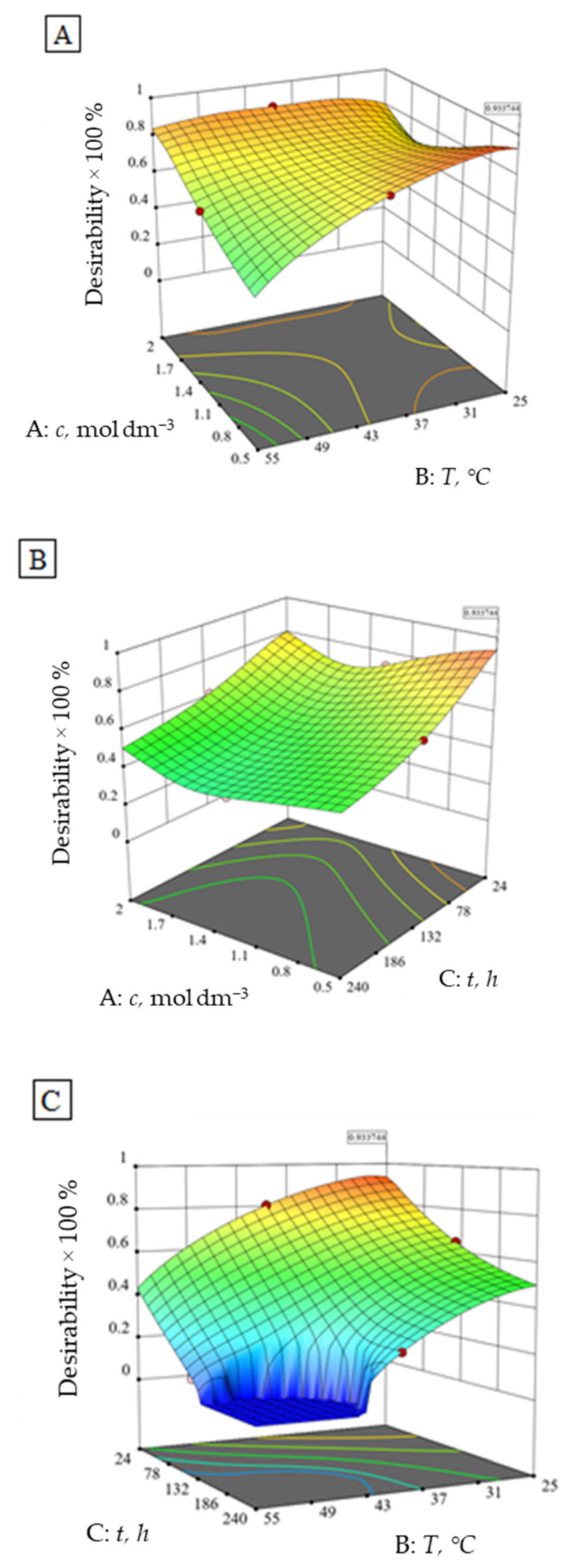
Desirability function of the number of eluted ions and alumina ceramics density independent of the (**A**) HNO_3_ concentration and temperature at constant time (132 h), (**B**) time and HNO_3_ concentration at constant temperature (40 °C) and (**C**) time and temperature at a constant HNO_3_ concentration (1.25 mol dm^−3^).

Optimum conditions, to achieve the least possible ion elution and highest alumina ceramics density, were found to be at the very beginning of the experiment (0.50 mol dm^−3^ HNO_3_, 25 °C, 24 h) with a desirability of 93% (Figure 5). The value of the 93% desirability function means that 93% of the maximum response value is achieved considering the given constraints and criteria.

A confirmatory experiment was conducted with the obtained parameters that are not covered by Box–Behnken design (vertex of the cube) [19]. The actual and predicted values were compared (Table 8) and a small deviation is present. However, the verification shown in Table 9 indicates that the obtained models may be considered adequate for the prediction of the alumina ceramics corrosion resistance optimum.

Furthermore, a second optimum may be defined at 2.00 mol dm^−3^ HNO_3_, 40 °C, 24 h with a desirability of 87 %, according to the numerical and graphical optimization (Figure 5). The results of the conducted confirmatory test in the second optimum are within 95 % of the confirmation interval (Table 9).

The first optimum is to be expected to a certain extent, while the second optimum is probably as a consequence of the lower impact of the higher HNO_3_ concentration at higher temperatures on the investigated alumina. The plateau visible in Figure 5C represents the experimental area that does not satisfy the desirability conditions.

## 4. Summary and Conclusions

In this study, the chemical stability of alumina was investigated at 25, 40 and 55 °C and HNO_3_ concentrations of 0.50, 1.25 and 2.00 mol dm^−3^ in a time frame of up to 240 h. The experiment was conducted according to the Box–Behnken design in order to estimate the conditions at which maximum corrosion resistance was achieved.

Regression models showed a higher elution of ions from alumina ceramics at a lower concentration of HNO_3_ and higher temperatures with time. Consequently, at the mentioned conditions, lower alumina ceramics density values were measured.

Within the experimental “sample-corrosive media” area, optimum conditions for reaching the highest corrosion resistance, i.e., the lowest number of eluted ions and the highest alumina ceramics density were achieved after the minimum exposure time (24 h) to 0.50 mol dm^−3^ HNO_3_ at 25 °C. Furthermore, a second optimum was present at 2.00 mol dm^−3^ HNO_3_, 40 °C, 24 h, but with a lower desirability. Lower HNO_3_ concentrations at higher temperatures were shown to be more influential on the dissolution of segregated impurities (CaO, Fe_2_O_3_, Na_2_O and SiO_2_) and sintering aid (MgO) in the grain boundaries of the alumina ceramics than the higher HNO_3_ concentrations.

## Figures and Tables

**Table 1:** Chemical composition of the alumina used in this research.

Component	Fe_2_O_3_	CaO	SiO_2_	MgO	Na_2_O	Al_2_O_3_
wt%	0.018	0.02	0.0325	0.045	0.05	balance

**Table 2:** Factors used in the Box–Behnken design.

Independent Variable	−1 Level	0	+1 Level
*c* (HNO_3_), mol dm^−3^	0.50	1.25	2.00
*T*, °C	25	40	55
*t*, h	24	132	240

**Table 3:** Design points.

No	*c* (HNO_3_), mol dm^−3^	*T*, °C	*t*, h
1	1.25	25	240
2	1.25	40	132
3	2.00	40	24
4	2.00	55	132
5	0.50	40	24
6	2.00	40	240
7	1.25	40	132
8	1.25	40	132
9	0.50	25	132
10	1.25	40	132
11	1.25	40	132
12	1.25	25	24
13	0.50	40	240
14	1.25	55	240
15	2.00	25	132
16	0.50	55	132
17	1.25	55	24

**Table 4:** Properties of sintered Al_2_O_3_ samples: density, hardness (HV1) and fracture toughness.

Sample	*ρ*, g cm^−3^	*HV*1	*K*_IC_, MPa m^1/2^
Al_2_O_3_	3.864 ± 0.018	1762 ± 77	5.44 ± 0.93

**Table 5:** ANOVA for the amount of eluted Al^3+^ ions from Al_2_O_3_ ceramics after exposure to HNO_3_.

Source	Sum of Squares	df	Mean Square	*F*-Value	*p*-Value (Prob > *F*)
Model	1.1658	9	0.1295	1092.97	<0.0001
A-Concentration	0.0013	1	0.0013	10.57	0.0140
B-Temperature	0.1513	1	0.1513	1276.89	<0.0001
C-Time	0.4586	1	0.4586	3869.36	<0.0001
AB	0.0198	1	0.0198	167.35	<0.0001
B^2^	0.0156	1	0.0156	131.21	<0.0001
C^2^	0.0064	1	0.0064	53.69	0.0002
A^2^B	0.0035	1	0.0035	29.36	0.0010
A^2^C	0.0047	1	0.0047	39.98	0.0004
AB^2^	0.0325	1	0.0325	274.38	<0.0001
Residual	0.0008	7	0.0001		
Lack of Fit	0.0004	3	0.0001	1.0153	0.4737 *
Pure Error	0.0005	4	0.0001		
Cor Total	1.1666	16			

* not significant; *α* = 0.05; *R*^2^ = 0.999.

**Table 6:** All experimental values used for the response surface plots and regression equations.

Run	*c*, mol dm^−^^3^	*T*, °C	*t*, h	µg (Al^3+^) cm^−^^2^	µg (Ca^2+^) cm^−^^2^	µg (Fe^3+^) cm^−^^2^	µg (Mg^2+^) cm^−^^2^	µg (Na^+^) cm^−^^2^	*ρ*, g cm^−^^3^
1	2.00	40	240	1.992	2.832	0.013	0.233	0.540	3.865
2	2.00	25	132	1.023	1.455	0.007	0.127	0.253	3.855
3	1.25	25	24	0.461	0.674	0.007	0.045	0.065	3.844
4	1.25	40	132	1.352	1.970	0.050	0.384	0.337	3.871
5	0.50	25	132	1.347	1.922	0.039	0.149	0.237	3.884
6	1.25	55	240	3.045	4.354	0.061	0.409	0.739	3.852
7	1.25	55	24	1.102	1.611	0.032	0.074	0.183	3.854
8	0.50	40	240	2.094	3.006	0.057	0.335	0.685	3.872
9	0.50	55	132	2.583	3.700	0.108	0.389	0.611	3.855
10	1.25	40	132	1.392	2.101	0.047	0.346	0.363	3.867
11	2.00	55	132	1.383	1.961	0.011	0.166	0.404	3.864
12	0.50	40	24	0.752	1.048	0.012	0.131	0.192	3.874
13	1.25	40	132	1.396	2.076	0.048	0.348	0.345	3.867
14	1.25	40	132	1.414	2.133	0.050	0.350	0.354	3.862
15	1.25	25	240	1.790	2.442	0.025	0.196	0.445	3.853
16	2.00	40	24	0.692	0.999	0.002	0.066	0.095	3.877
17	1.25	40	132	1.415	2.060	0.047	0.374	0.289	3.860

**Table 7:** Regression equations with coded factors for the number of eluted ions and density of Al_2_O_3_ ceramics.

Response	Regression Equations
Al^3+^	1.18 − 0.018A + 0.19B + 0.34C − 0.07AB + 0.061B^2^ − 0.039C^2^ − 0.042A^2^B − 0.049A^2^C − 0.13AB^2^
Ca^2+^	1.43 − 0.019A + 0.24B + 0.39C − 0.086AB + 0.057B^2^ − 0.062C^2^ − 0.06A^2^B − 0.042A^2^C − 0.16AB^2^
Fe^3+^	0.049 − 0.013A + 0.017B + 0.013C − 0.016AB − 8.213·10^−3^AC + 2.572·10^−3^ BC − 8.784·10^−3^ A^2^ − 0.019C^2^ − 0.019 AB^2^
Mg^2+^	0.60 − 0.056A + 0.069B + 0.13C − 0.047AB + 0.034BC − 0.064A^2^ − 0.093B^2^ − 0.12C^2^
Na^+^	0.35 − 0.054A + 0.12B + 0.23C − 0.056AB + 0.044BC + 0.031A^2^
*ρ*	3.86 − 0.003A + 2.2·10^−3^ B + 9.5·10^−3^ AB + 0.01 A^2^ − 0.011 B^2^ − 0.007A^2^B

A—*c* (HNO_3_), mol dm^−3^; B—*T*, °C; C—*t*, h.

**Table 8:** Verification of experimentally and calculated values for the number of eluted ions and Al_2_O_3_ ceramics density at randomly chosen parameters of corrosion.

No of Verification	Response	Experimental Values	Predicted Values	Low CI (95%)	High CI (95%)
1	Experimental parameters: 0.50 mol dm^−^^3^ HNO_3_, 25 °C, 132 h				
	µg (Al^3+^) cm^−^^2^	1.347	1.351	1.293	1.409
	µg (Ca^2+^) cm^−^^2^	1.922	1.947	1.786	2.115
	µg (Fe^3+^) cm^−^^2^	0.039	0.040	0.034	0.046
	µg (Mg^2+^) cm^−^^2^	0.148	0.147	0.114	0.185
	µg (Na^+^) cm^−^^2^	0.237	0.258	0.212	0.304
	*ρ*, g cm^−^^3^	3.884	3.880	3.870	3.891
2	Experimental parameters: 0.50 mol dm^−^^3^ HNO_3_, 40 °C, 240 h				
	µg (Al^3+^) cm^−^^2^	2.094	2.098	2.036	2.160
	µg (Ca^2+^) cm^−^^2^	3.006	3.013	2.841	3.191
	µg (Fe^3+^) cm^−^^2^	0.057	0.056	0.050	0.062
	µg (Mg^2+^) cm^−^^2^	0.335	0.367	0.323	0.415
	µg (Na^+^) cm^−^^2^	0.685	0.665	0.630	0.701
	*ρ*, g cm^−^^3^	3.872	3.877	3.870	3.883
3	Experimental parameters: 1.25 mol dm^−^^3^ HNO_3_, 25 °C, 240 h				
	µg (Al^3+^) cm^−^^2^	1.790	1.810	1.754	1.868
	µg (Ca^2+^) cm^−^^2^	2.442	2.473	2.318	2.635
	µg (Fe^3+^) cm^−^^2^	0.025	0.024	0.019	0.029
	µg (Mg^2+^) cm^−^^2^	0.196	0.176	0.140	0.217
	µg (Na^+^) cm^−^^2^	0.445	0.420	0.374	0.465
	*ρ*, g cm^−^^3^	3.853	3.850	3.843	3.858
4	Experimental parameters: 1.25 mol dm^−^^3^ HNO_3_, 55 °C, 24 h				
	µg (Al^3+^) cm^−^^2^	1.102	1.118	1.073	1.163
	µg (Ca^2+^) cm^−^^2^	1.611	1.636	1.511	1.768
	µg (Fe^3+^) cm^−^^2^	0.032	0.032	0.027	0.037
	µg (Mg^2+^) cm^−^^2^	0.074	0.088	0.063	0.118
	µg (Na^+^) cm^−^^2^	0.183	0.186	0.140	0.231
	*ρ*, g cm^−^^3^	3.854	3.855	3.847	3.863
5	Experimental parameters: 2.00 mol dm^−^^3^ HNO_3_, 40 °C, 24 h				
	µg(Al^3+^) cm^−^^2^	0.692	0.694	0.659	0.730
	µg (Ca^2+^) cm^−^^2^	0.999	1.004	0.906	1.108
	µg (Fe^3+^) cm^−^^2^	0.002	0.004	0.002	0.010
	µg (Mg^2+^) cm^−^^2^	0.066	0.055	0.039	0.075
	µg (Na^+^) cm^−^^2^	0.095	0.089	0.053	0.125
	*ρ*, g cm^−^^3^	3.877	3.871	3.864	3.877

**Table 9:** Verification of experimentally and calculated values of the number of eluted ions and Al_2_O_3_ ceramics density at the assessed optimal corrosion parameters.

No of Verification	Response	Experimental Values	Predicted Values	Low CI (95%)	High CI (95%)
1	Experimental parameters: 0.50 mol dm^−3^ HNO_3_, 25 °C, 24 h, desirability 93%				
	µg (Al^3+^) cm^−^^2^	0.512	0.695	0.649	0.742
	µg (Ca^2+^) cm^−^^2^	0.724	0.970	0.845	1.106
	µg (Fe^3+^) cm^−^^2^	0.013	0.003	0.005	0.010
	µg (Mg^2+^) cm^−^^2^	0.046	0.030	0.014	0.053
	µg (Na^+^) cm^−^^2^	0.058	0.068	0.010	0.126
	*ρ*, g cm^−^^3^	3.868	3.880	3.870	3.891
2	Experimental parameters: 2.00 mol dm^−^^3^ HNO_3_, 40 °C, 24 h, desirability 87%				
	µg (Al^3+^) cm^−^^2^	0.692	0.694	0.659	0.730
	µg (Ca^2+^) cm^−^^2^	0.999	1.004	0.906	1.108
	µg (Fe^3+^) cm^−^^2^	0.002	0.004	0.002	0.010
	µg (Mg^2+^) cm^−^^2^	0.066	0.055	0.039	0.075
	µg (Na^+^) cm^−^^2^	0.095	0.089	0.053	0.125
	*ρ*, g cm^−^^3^	3.866	3.871	3.864	3.877

## Data Availability

Data sharing is not applicable to this article.
